# Expression of the TIGIT axis and the CD39/CD73 purinergic pathway in bone metastasis-derived immune cells

**DOI:** 10.1007/s00262-025-04030-2

**Published:** 2025-04-24

**Authors:** Elias Brauneck, Leon-Gordian Leonhardt, Anne Marie Assemissen, Yagana Wahid, Moritz Kruppa, Niklas Kruppa, Julius Krüger, Stephan Menzel, Friedrich Koch-Nolte, Julian Kylies, Katja Weisel, Carsten Bokemeyer, Jasmin Wellbrock, Walter Fiedler, Lennart Viezens, Franziska Brauneck

**Affiliations:** 1 Division of Spine Surgery, Department of Trauma and Orthopedic Surgery, University Medical Center Hamburg-Eppendorf, Hamburg, Germany; 2 Department of Oncology, Hematology and Bone Marrow Transplantation with Section Pneumology, Hubertus Wald University Cancer Center, University Medical Center Hamburg-Eppendorf, Hamburg, Germany; 3 Mildred Scheel Cancer Career Center HaTriCS4, University Medical Center Hamburg-Eppendorf, Hamburg, Germany; 4 Institute of Immunology, University Medical Center Hamburg-Eppendorf, Hamburg, Germany; 5 Core Facility Nanobodies, University of Bonn, Venusberg-Campus 1, 53127 Bonn, Germany

**Keywords:** Bone metastasis, Immune cells, Anti-tumor immunity, Checkpoint molecules, TIGIT axis, Purinergic signaling

## Abstract

**Background:**

Bone metastases (BM) represent one of the most common sites of metastasis. The study aimed to compare the composition of immune cell infiltration from aspirates of different BM prior to systemic therapy.

**Method:**

Phenotypic and functional analyses were conducted via multiparametric flow cytometry (MFC) on BM-derived aspirates obtained from patients with breast cancer (BC, *n* = 6), patients with prostate cancer (PC, *n* = 5), patients with non-small-cell lung cancer (NSCLC) (*n* = 7), patients with myeloma (MM, *n* = 10) and bone aspirates from age-matched non-malignant controls (NMC, *n* = 10).

**Results:**

Across all tumors aspirates the fraction of CD8^+^ T cells was reduced. In contrast, infiltration by immunosuppressive CD56^+^CD16^−^NK and CD163^+^CD86^+^ M2-like macrophages was increased in BM compared to NMC aspirates. BM-derived CD8^+^ T cells aberrantly co-expressed TIGIT with PVRIG or CD39. Similarly, BM-derived cytotoxic NK cells co-expressed TIGIT and PVRIG. In addition, BM-derived M2-like macrophages exhibited an increased subset of cells co-expressing either TIGIT and PVRL4 or CD112 and CD155. Using a myeloma model, functional in vitro studies showed that blockade of TIGIT and CD39 leads to increased PBMC-mediated lysis of myeloma cells.

**Conclusion:**

The study shows that an altered immune cell composition is present in BM across the different tumor entities. Additionally, molecules of the TIGIT checkpoint as well as of the purinergic pathway are aberrantly expressed by BM-infiltrating CD8^+^ T cells, NK cells and macrophages and also functionally relevant for tumor cell lysis.

**Supplementary Information:**

The online version contains supplementary material available at 10.1007/s00262-025-04030-2.

## Introduction

Bone metastases (BM) represent one of the most prevalent site of metastasis across all cancer types [1]. Over 80% of patients with advanced breast cancer (BC), prostate cancer (PC), and 30–40% of lung cancer patients develop BM [2]. Recently the presence of BM has been identified as an independent predictor of shorter overall survival (OS) and progression-free survival (PFS) in various cancers, including BC, PC, and lung cancer [3]. Furthermore, the occurrence of BM significantly impacts patients` quality of life due to severe pain, impaired mobility, pathologic fractures, spinal cord compression, bone marrow aplasia, and hypercalcemia [4].

Regarding novel therapeutic options, immune oncology represents a rapidly developing field with the prospect of positively influencing the prognosis of malignant tumors [5–7]. Over the past decade, immune checkpoint inhibitors (ICIs) have demonstrated impressive response rates and OS benefits in the treatment of many metastatic malignancies [8]. Particularly ICIs targeting programmed cell death protein 1 (PD-1), its ligand PD-L1 or cytotoxic T lymphocyte-associated protein 4 (CTLA-4), have achieved extraordinary success in treating cancers such as non-small cell lung cancer (NSCLC) [8]. Nevertheless, a noticeable percentage of cancer patients exhibit either primary or acquired resistance to ICIs [9]. The underlying mechanisms are still insufficiently understood. Factors known to positively influence the immune response include high level of microsatellite instability (MSI-H), defects in a mismatch repair gene (dMMR), high tumor mutational burden (TMB-H), immune cell infiltration and level of checkpoint expression [10]. Importantly, the site of metastasis may also determine ICI efficacy as demonstrated in recent studies including bone and liver metastases [11, 12]. It has been demonstrated that the bone-specific response in BM correlates with survival outcomes in patients treated with ICIs. Achievement of a bone-specific objective response was associated with a longer OS and PFS in NSCLC patients [13]. Regarding bone tumors and skeletal metastases, it was recently demonstrated that combined treatments with denosumab associates with an increased abundance of T cells in the tumor microenvironment (TME) and increased amount of anti-tumor cytokines (such as interferon-gamma). Receptor Activator of Nuclear Factor Kappa B and its ligand (RANKL/RANK) has been demonstrated to modulate diverse facets of the immune reaction, including regulatory T cells, CD8 ^+^ and CD4 ^+^ T cells and myeloid suppressor cells [14].

Recent findings suggest that bone turnover markers, particularly P1NP and CTX, reflect not only osteoblast/osteoclast activity but also immune modulatory changes in BM, even when the primary site is unknown [15]. Elevated levels often indicate extensive skeletal disease. Shifts in these markers during bisphosphonate or denosumab therapy may reflect treatment efficacy [15].

Although the immunologic mechanisms are not fully understood, a specific immunosuppressive microenvironment may enable BM to become resistant to ICIs [16].

In addition to PD-1/PD-L1 and CTLA4 advancements in targeted immune checkpoint blockade and the identification and evaluation of new target molecules are needed. The present study investigated newly discovered molecules of two promising immunological pathways: the T cell immunoreceptor with immunoglobulin and ITIM domain (TIGIT) axis and the purinergic pathway that degrades ATP into immunosuppressive adenosine [17, 18]. The inhibitory receptor TIGIT competes together with the inhibitory receptor PVRIG (poliovirus receptor-related immunoglobulin) against the activating receptor CD226 (DNAX accessory molecule-1, DNAM-1) for their shared ligands including CD112 (poliovirus receptor-related 2, PVRL2), and CD155 (poliovirus receptor, PVR) which are expressed on cancer cells and antigen-presenting cells [19–22]. TIGIT also binds poliovirus receptor-related protein 4 (PVRL4 or Nectin-4), a TIGIT-specific ligand recently known to be expressed on tumor cells. Together, TIGIT/PVRIG—CD112/CD155/PVRL4 signaling has recently shown to prevent T cell proliferation, tumor cell lysis, and the production of proinflammatory cytokines [21, 23, 24]. TIGIT was originally described as an inhibitory receptor of T cells, but we and others recently detected its expression also on tumor-associated NK cells and macrophages [25–27].

Beside the classical co-regulatory signaling, the metabolic ATP-adenosine pathway has also proven to be a promising target for novel immunotherapeutic strategies: Extracellular adenosine regulates both innate and adaptive immunity via immunosuppressive signaling that depends on the respective adenosine receptors [28]. Extracellular adenosine is produced by the degradation of adenosine triphosphate (ATP) by the ectonucleotidases CD39/CD73 [29]. It has already been shown that extracellular adenosine as well as the two ectonucleotidases are prevalent in several types of cancer and the TME [18]. Expression of CD39 in CD8 ^+^ T cells is associated with T cell exhaustion in the primary tumors as well as in the peripheral blood [30]. Whereas expression of CD73 was predominantly found on tumor cells in comparison with effector CD8^+^ T cells [31, 32]. Interestingly, we and others previously found that the expression of the TIGIT pathway is closely linked to that of CD39 and CD73 during immune cell exhaustion [30, 33].

BC and NSCLC are among the most common cancers in women, and PC and NSCLC are among the most common cancers in men [34]. Yet ICIs are only approved for triple-negative BC and NSCLC [35]. However, for multiple myeloma (MM) a variety of immunotherapeutic strategies have already being successfully approved or are under clinical testing [36].

In this study, we systematically compared the immune profile of BM aspirates from untreated patients with advanced malignancies (BC, PC, NSCLC) or untreated MM to aspirates from a NMC of comparable age. We hypothesized that tumor-infiltrated bone marrow exhibits immunosuppressive features impeding immunotherapeutic efficacy. Therefore, we assessed T cell, NK cell, and macrophage populations alongside key regulatory molecules (TIGIT, PVRIG, CD112, CD155, PVRL4, CD39, and CD73). By identifying shared and distinct alterations across different tumor entities, our goal was to reveal novel immunomodulatory pathways and inform future individualized immune-based interventions for BM.

## Methods

### Clinical cohorts

BM-derived aspirates were obtained from patients with BC (*n* = 6), PC (*n* = 5), NSCLC (*n* = 7) and MM (*n* = 10). All patients included in this study had an initially suspected malignant spinal lesion, no prior history of an advanced malignancy or tumor-directed treatment and required surgical intervention due to unstable spinal osteolysis and impending or manifest neurological deficits. During spinal surgery, tissue samples were collected from the affected vertebral bodies for histopathological diagnosis as part of standard clinical practice. Additionally, bone marrow aspirates were obtained from the same vertebral bodies. Aspirates were collected from spinal BM during surgery after written informed consent in accordance with the Declaration of Helsinki and approval of the local ethics board (PV5119). Surgery was indicated for unstable spinal osteolysis with imminent or manifest neurological impairment (skeletal-related event). As a control, we analyzed bone marrow samples from a non-malignant control group (NMC, *n* = 10) that did not differ in age from the malignant cohort (*p* = 0.31) and confirmed to be free of malignant disease through histopathological assessment (PV2021-30031-WF). The mean age of the patient cohort (53.6% female) was 67.6 years (± 10.4) (Supplementary Table 1; Patient characteristics). The mean age of the non-malignant control cohort (60.0% female) was 70.5 years (± 31.0).

### Multiparametric flow cytometry

For multiparameter flow cytometry (MFC) analyses, cryopreserved bone marrow mononuclear cells from patients with BC, PC, NSCLC, and MM as well as NMCs were processed as recently described [26].

### Flow cytometry data analyses

Three panels per donor were stained, whereby in panel 1 primarily T cells were characterized, panel 2 analyzed NK cells and panel 3 investigated macrophages. Stained samples were measured by the BD FACSymphony™ A3 flow cytometer. The data set was analyzed using FlowJo software version 10.5.2 (Tree Star Inc., Ashland, OR, USA). In each experiment, mononuclear cells of NMCs were included as internal control. Instrument performance was verified using the Cytometer Setup & Tracking (CS&T) system (BD Biosciences), applying CST application settings to ensure comparable flow cytometry results over time. The threshold for positive staining was determined using unstained- or fluorescence minus one (FMO) control. To identify T cells, NK cells, and macrophages a sequential gating strategy for each flow panel was created (gating strategies are representatively demonstrated in the Supplementary Fig. 1–3).

### Statistical analysis

Statistical analyses were carried out using Prism 8.0 (GraphPad Software, San Diego, CA, USA). The normality of data distribution was analyzed using the Kolmogorov–Smirnov test. Parametric data were further compared using the t test for two groups and the ordinary one-way ANOVA test for comparisons of ≥ 2 groups. Nonparametric tests, including the Mann–Whitney U test and the Kruskal–Wallis test, were applied to data with non-normal distributions. Checkpoint correlation was assessed applying the Spearman´s rank correlation as these data did not adhere to normality. Frequencies in the text are described as medians unless stated otherwise (as indicated in the figure legend). *p*-values less than 0.05 were considered significant. Specifically, * indicates *p*-values between 0.01 and 0.05, ** indicates *p*-values between 0.001 and 0.01, *** indicates *p*-values between 0.0001 and 0.001, and **** indicates p values less than 0.0001.

### T-Distributed stochastic neighbor embedding analyses

*t*-Distributed stochastic neighbor embedding (tSNE) analyses were performed on aspirates from NMCs, patients with BC, PC, NSCLC, and MM, using the tSNE plugin in FlowJo 10.6.2. A subset of 3000–4000 events of the enrolled cell populations was randomly selected for individual donors and merged into an individual expression matrix. Within the multiparametric analyses, the following channels were excluded from the matrix in the 3 panels: viability, dump channel that contained CD19, and dependent of the tumor/panel type the tumor markers EpCAM, CD138, and the respective lineage markers CD16, CD56, CD14, offset, residual and time. The aim was to collect only the protein expression data of the molecules of interest for our tSNE analysis. Finally, cells were down sampled, concatenated, and exported to generate tSNE maps. As in our previous studies, a perplexity parameter of 30 and an iteration number of 550 were applied for dimensionality reduction. The generated matrix consists of two columns, those of tSNE dimension 1 and dimension 2.

### ATP titration assay

To test the functional relevance of the CD39 blockade, first the effects of the CD39 substrate the exogenous adenosine triphosphate (ATP) was analyzed on myeloma cell lines and peripheral blood mononuclear cell (PBMCs). The MM cell lines U266 and LP-1 as well as freshly isolated PBMCs of healthy controls were used. The effect of exogenous ATP on the viability was evaluated by MFC analyses measuring the expression of 7AAD over 24 h.

### PBMC-mediated cytotoxic assays

PBMC-mediated cytotoxicity was evaluated by testing several non-malignant donor PBMCs as effector cells and the MM cell lines U266 and TF-1 as target cells. PBMCs were co-cultured with MM cells, which were labeled with CellTracker™ (CT) green CMFDA (Invitrogen Thermo Fisher, Waltham, Massachusetts, USA) according to the manufacturer’s instructions in a 6:1 effector:target ratio. Co-culture was plated in a 96-well plate (1 × 10^6^ cells/mL) in R10 culture medium and incubated with 1 µg/ml Dynabeads™ Human T-Activator CD3/CD28 (Thermo Fisher Scientific, Vilnius, Lithuania) with different blocking antibodies or nanobodies for up to 72 h at 37 °C and 5% CO_2_. Each well of the 96-well plate contained a 200 µL volume in total, including the blocking antibodies. For experiments with single blockade of TIGIT, co-cultured cells were incubated with 50 µg/mL anti-TIGIT antibody (Ultra-LEAF™ Purified anti-human TIGIT antibody clone A15153G, BioLegend, San Diego, CA, USA) or mouse IgG2a isotype control (Ultra-LEAF™ Purified Mouse IgG2a, _K_ Isotype Ctrl antibody, clone MG2a-53, BioLegend, San Diego, CA, USA) or left untreated. For the evaluation of the CD39 blockade 100 µg/mL control nanobody (the target non-binding nanobody L-10e was used in the same format as the CD39-specific SB24 nanobody). The inhibitory anti-CD39 nanobody SB24 was generated from an immunized alpaca and reformatted into a human IgG1 heavy-chain antibody format carrying the LALAPG mutations using established protocols [37, 38]. After incubation, the cells of each well were collected, washed and stained with 7-Aminoactinomycin D (7-AAD) Viability Staining Solution (BioLegend, San Diego, CA, USA) according to the manufacturer’s protocol. The samples were subsequently analyzed on a BD FACSymphony™ A3 with BD FACSDiva software version 8 (BD Biosciences, Franklin Lakes, NJ, USA). The frequency of MM cell lysis was determined via positivity of 7-AAD and CT green using flow cytometry.

## Results

### Immune cell infiltration in bone metastasis differs across tumor entities

The present study characterized selected immune cell populations of BM aspirates of 28 donors (BC *n* = 6, PC *n* = 5, NSCLC *n* = 7, and MM *n* = 10; Supplementary Table 1). As control, non-malignant bone aspirates of patients with excluded malignancies (NMC *n* = 10) were analyzed. MFC analyses of 3 panels were assessed to clearly distinguish T cells, NK cells and macrophages. By adding a dump channel irrelevant cell populations were excluded, respectively. Within the tubes, CD3^+^ T cells were differentiated into CD8^+^/CD4^+^ T cells, total NK cells into CD56^+^CD16^−^, CD56^+^CD16^+^ and CD56^−^CD16^+^ and M0 macrophages were differentiated into M2-like CD163^+^CD86^+^ and M1-like CD163^−^CD86^+^ sub-populations.

**Fig. 1:**
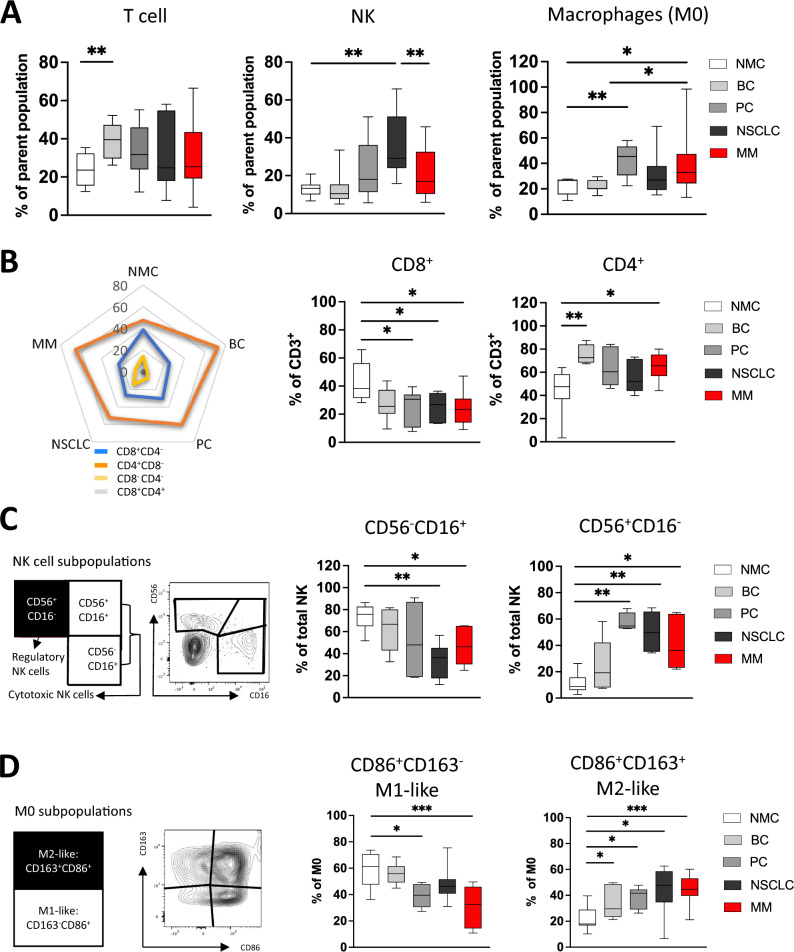
Immune cell infiltration differs in bone metastasis of patients with breast cancer, prostate cancer, non-small cell lung cancer, and multiple myeloma. Multiparametric flow cytometry (MFC) analyses of CD3^+^ T cells, NK cells, and macrophages were performed using 3 MFC panels. Bone aspirates from patients with untreated breast cancer (BC *n* = 6), prostate cancer (PC *n* = 5), non-small cell lung cancer (NSCLC *n* = 7) and multiple myeloma (MM *n* = 10) were analyzed. The malignant aspirates were compared to bone aspirates from age-matched non-malignant patients undergoing spine surgery (NMC *n* = 10). **A** Summary data compare the infiltration of CD3^+^ T cells, NK cells and macrophages in the bone aspirates. **B** Spider plots and summary data show CD3^+^ T cell sub-populations based on the CD8 and CD4 expression status. **C** Graphical illustration and summary data show the distribution of NK cell sub-populations based on CD56 and CD16 status. **D** Graphical illustration, exemplary FACS plots and summary data compare the (M1-like) CD86^+^CD163^−^ and suppressive (M2-like) CD163^+^CD86^+^ macrophages (CD68^+^CD14^+^). Frequencies are displayed with the median. *p* Values were obtained by the ANOVA test. *p < 0.05, **p < 0.01, ***p < 0.001, ****p < 0.0001

Within the cohorts, infiltration of immune cells varied between the tumor entities. In BC-derived aspirates, an increased frequency of CD3^+^ T cells was observed compared to the NMC cohort. The frequency of NK cells was increased in NSCLC aspirates in comparison with NMCs as well as MM patients. BM aspirates of PC and MM patients contained a higher infiltration rate of macrophages compared to that found in NMCs (Fig. 1A). Representative MFC gating strategies for the 3 MFC panels to detect the respective immune cell populations are illustrated in the Supplementary Fig. 1–3.

Further sub-analyses of CD3^+^ T cells revealed that in PC, NSCLC, and MM aspirates the frequency of CD8^+^ T cells was significantly reduced compared to that in NMC aspirates. Additionally, our study showed an increased CD4^+^ T cell fraction in all 4 tumor entities, reaching statistical significance in BC and MM patient aspirates (Fig. 1B). In all groups, CD4^−^CD8^−^ and CD4^+^CD8^+^ T cells constituted only a small proportion of the total CD3^+^T cell compartment (Fig. 1B and Supplementary Fig. 4A).

Phenotypic NK cell differentiation was further conducted based on the categorization into CD56^+^CD16^−^ NK cells as these exert primarily regulatory effects and into the cytotoxic CD56^−^CD16^+^ and CD56^+^CD16^+^ NK cells (Fig. 1C) [39]. Recent research suggests that the two cytotoxic subsets are comparable in antibody-dependent cellular cytotoxicity but differ in their degranulation capacity against target cells [40, 41]. The frequency of cytotoxic CD56^−^CD16^+^ NK cell subset was similar between in the non-malignant and malignant groups, except in NSCLC- and MM-derived aspirates, where they were reduced (Fig. 1C). For the CD56^+^CD16^+^ NK cell subset no significant differences occurred (Supplementary Fig. 4B). In contrast, the frequencies of regulatory CD56^+^CD16^−^ NK cells were significantly increased in aspirates of PC, NSCLC and MM patients in comparison with that of NMCs (Fig. 1C).

As macrophages can be roughly differentiated into more immunosuppressive (M2-like) and inflammatory (M1-like) acting subsets this was further investigated based on expression of CD163 and CD86. Compared to NMCs the frequency of CD86^+^CD163^−^ M1-like macrophages appeared to be reduced in PC and MM patient aspirates. In contrast, the fraction of CD163^+^CD86^+^ M2-like macrophages was significantly increased in all 4 of 4 tumor entities compared to that seen in NMC aspirates (Fig. 1D).

Across the tumor entities, BM aspirates contained a reduced fraction of CD8^+^ T cells and a higher infiltration of immunosuppressive NK and macrophages compared to the NMC controls. No characteristic difference was found between aspirates of solid tumors versus MM.

### Bone metastasis-derived CD8^+^ T cells co-express TIGIT with PVRIG and CD39

Aberrant expression of co-regulatory receptors including PD-1, TIGIT, and CD39 has been recently identified as sign of CD8^+^ T cell dysfunction in TILs of primary tumor tissue [42]. By applying MFC this study analyzed expression of the pre-selected checkpoint molecules TIGIT, PVRIG, CD226, CD39 and CD73 in CD8^+^ and CD4^+ ^T cells of BM aspirates. Fluorescence minus one (FMO) controls were applied to differentiate between specific and background signal.

**Fig. 2:**
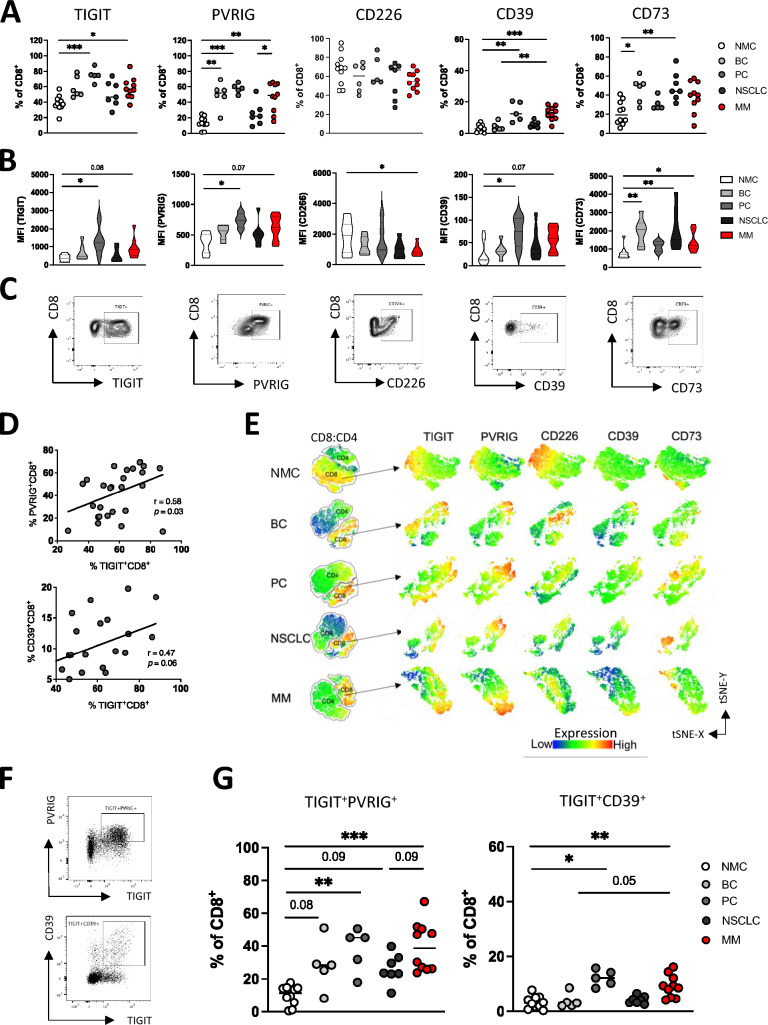
Bone metastasis-derived CD8^+^ T cells co-express TIGIT with PVRIG and CD39. Expression of TIGIT, PVRIG, CD226, CD39 and CD73 was analyzed on CD8^+^ T cells. Multiparametric flow cytometry (MFC) analyses included bone aspirates from bone metastases (BM) of patients with breast cancer (BC *n* = 6), patients with prostate cancer (PC *n* = 5), patients with non-small cell lung cancer (NSCLC *n* = 7), and patients with multiple myeloma (MM *n* = 10). The malignant aspirates were compared to bone aspirates from age-matched non-malignant patients undergoing spine surgery (NMC *n* = 10). **A** Frequencies of TIGIT, PVRIG, CD226, CD39 and CD73 expression on indicated CD8^+^ T cells. **B** Median fluorescence intensity (MFI) of the respective target proteins on indicated CD8^+^ T cells. **C** Exemplary FACS plots illustrate target protein expression on CD8^+^ T cells. **D** Correlation of TIGIT with PVRIG expression as well as with CD39 on CD8^+^ T cells is depicted including all malignant aspirates. **E** T-distributed stochastic neighbor embedding (tSNE) heat maps illustrate the expression and distribution of TIGIT, PVRIG, CD226, CD39 and CD73 on CD8^+^ T cells from aspirates of patients with BC, PC, NSCLC and MM (*n* = 4, respectively). **F** Exemplary flow plots illustrate co-expression pattern of PVRIG and CD39 with TIGIT on CD8^+^ T cells. **G** Summary data show the co-expression of TIGIT with PVRIG and CD39 on CD8^+^ T cells. Frequencies are displayed by the medians. *p* Values were obtained by the ANOVA and Kruskal Wallis test. Pearson’s test was used to test for correlations. *p < 0.05, **p < 0.01, ***p < 0.001, ****p < 0.0001

The present analyses revealed a heterogeneous expression status across the different entities, with relatively large expression variances. In comparison with NMCs, CD8^+^ T cells from BC aspirates showed a higher frequency of PVRIG^+^ and CD73^+^ cells, PC-derived CD8^+^ T cells displayed an increased fraction of TIGIT^+^, PVRIG^+^ and CD39^+^ cells, NSCLC-derived CD8^+^ T cells expressed more frequently CD73 on their surface, and MM-derived CD8^+^ T cells more frequently expressed TIGIT, PVRIG, CD39 and CD73 (Fig. 2A). No differences were found for the CD226 expression. These expression patterns were also evident in the evaluation of the median fluorescence intensity (MFI) (Fig. 2B). Exemplary target expression of the respective molecules is demonstrated in Fig. 2C. As no significant differences in expression were observed in bulk CD4^+^ T cells compared to NMCs, subsequent analyses were focused on CD8^+^ T cells (Supplementary Fig. 5).

Interestingly, there was a correlation between the expression status of TIGIT with PVRIG and with CD39 on CD8^+^ T cells in all BM aspirates (due to the small number of cases, the malignant cohorts were combined here, Fig. 2D). To further map the protein expression, tSNE analyses were conducted, revealing that TIGIT, PVRIG, and CD39 but not CD73 were expressed in the same subsets of BM-derived CD8^+^ T cells (Fig. 2E). Built on the tSNE results, individual co-expression of the respective molecules TIGIT, PVRIG, and CD39 was analyzed. As exemplary illustrated in Fig. 2F, in contrast to NMCs BM-aspirate derived CD8^+^ T cells more frequently co-expressed TIGIT and PVRIG across all tumor entities. Cross pathway, TIGIT and CD39 were more frequently co-expressed in BM aspirates of PC and MM patients (Fig. 2G).

In conclusion at least one target molecule of the TIGIT/PVRIG pathway axis or the CD39/CD73 purinergic pathway was more frequently and at a higher MFI expressed in each tumor entity (BC: PVRIG, CD73; PC: TIGIT, CD39; NSCLC: CD73; MM: TIGIT, PVRIG, CD39). No clear differences were found between BM aspirates from solid tumors and MM. Moreover, characteristic co-expression patterns were observed in BM. TIGIT was aberrantly co-expressed with PVRIG (4 of 4 tumor entities) and CD39 (2 of 4 tumor entities). In the context of CD8^+^ T cell dysfunction during tumor progression these expression patterns could indicate a functional relevance, and perhaps also synergistic effects of the two pathways.

### Bone metastasis-derived NK cells show a difference in the expression of the TIGIT pathway and purinergic signaling based on their functional subtype

Based on the shift of regulatory and cytotoxic NK cells subsets in the BM we further analyzed the checkpoint profile on these NK cells. It has been demonstrated that NK cells can express a variety of activating and inhibitory receptors, resulting in complex interactions with target cells. In dysfunctional NK cells from patients with tumor disease, a predominance of inhibitory receptors has been recently observed [27]. To further validate, if BM-derived NK cells also display an aberrant checkpoint profile, again expression of TIGIT, PVRIG, CD226, and of the ectonucleotidases CD39 and CD73 was analyzed by MFC on the respective NK cell panel. Expression analyses were also conducted on NK sub-populations (CD56^+^CD16^−^, CD56^+^CD16^+^ and CD56^−^CD16^+^). Fluorescence minus one (FMO) controls were used to determine the positive protein expression.

**Fig. 3:**
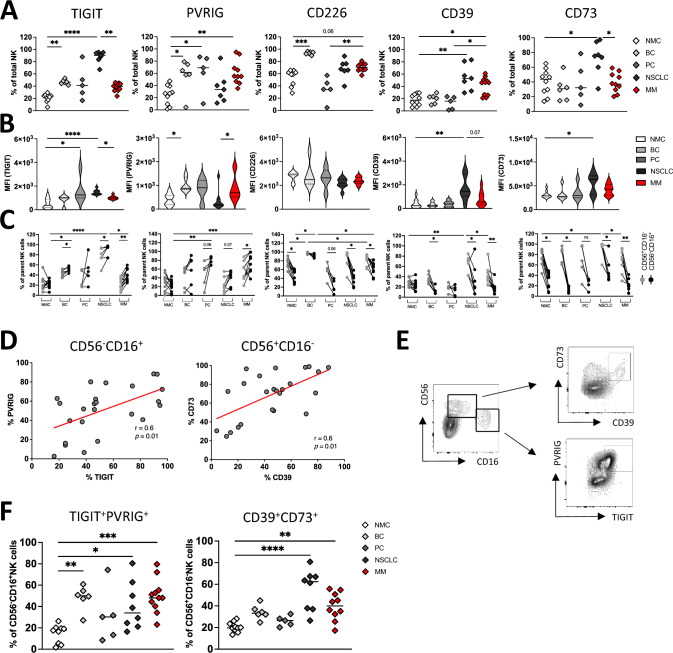
Bone metastasis-derived NK cells show a difference in the expression of the TIGIT pathway and purinergic signaling based on their functional subtype. Expression of TIGIT, PVRIG, CD226, and the ectonucleotidases CD39 and CD73 on NK cells was evaluated. As described, bone aspirates from patients with breast cancer (BC *n* = 6), patients with prostate cancer (PC *n* = 5), patients with non-small cell lung cancer (NSCLC *n* = 7), and patients with multiple myeloma (MM *n* = 10) were analyzed by multiparametric flow cytometry and compared to age-matched mononuclear cells of bone aspirates from non-malignant patients undergoing spine surgery (NMC *n* = 10). **A** Frequencies of TIGIT, PVRIG, CD266, CD39, and CD73 expression on indicated NK cells. **B** Median fluorescence intensity (MFI) of the respective target proteins on indicated NK cells. **C** Expression of the target proteins was compared between cytotoxic CD56^−^CD16^+^ and regulatory CD56^+^CD16^−^ NK cell sub-populations. **D** Correlation of TIGIT with PVRIG, and of CD39 with CD73 on CD56^−^CD16^+^ and regulatory CD56^+^CD16^−^ NK cells. **E** Exemplary flow plots illustrate the co-expression of PVRIG with TIGIT within the CD56^+^CD16^−^ NK cell subset and of CD39 with CD73 within the CD56^−^CD16^+^ NK cell subset. **F** Summary data displaying target protein co-expression on respective NK cell subsets. Frequencies are displayed with the medians. *p* Values were obtained by the ANOVA and Kruskal Wallis test. Pearson’s test was used to test for correlations. *p < 0.05, **p < 0.01, ***p < 0.001, ****p < 0.0001

In comparison with NMC aspirates, highest frequencies of TIGIT^+^ and CD226^+^ NK cells were detected in the aspirates of patients with BC and NSCLC. PVRIG showed a different expression pattern: In addition to BC aspirates, PC and MM aspirates contained higher frequencies of PVRIG^+^ NK cell subsets compared to NMC aspirates (Fig. 3A). These distribution patterns were also evident in the analyses of median fluorescence intensity (MFI, Fig. 3B). Regarding the purinergic pathway, CD39 was more frequently expressed on total NK cells of NSCLC and MM aspirates, while CD73 was mainly expressed on NK cells of NSCLC aspirates in comparison with NMCs.

Regarding the functional NK cell subset stratification, the study showed that both inhibitory receptors TIGIT and PVRIG were more frequently expressed by the cytotoxic CD56^−^CD16^+^ NK cell subset, while CD226 and the two metabolic enzymes CD39 and CD73 were dominantly expressed by the regulatory CD56^+^CD16^−^ NK cell subset (Fig. 3C). Correlation analyses revealed an association between expression of TIGIT and PVRIG on CD56^−^CD16^+^ NK cells and between CD39 and CD73 but not CD226 on CD56^+^CD16^−^ NK cells (due to the small number of cases, the malignant cohorts were combined here again, Fig. 3D). Subsequent co-expression analyses within the CD56^−^CD16^+^ and the CD56^+^CD16^−^ NK cell subset were conducted. As exemplarily shown in Fig. 3E, a significant co-expression pattern of TIGIT and PVRIG was detected in the CD56^−^CD16^+^NK cells and of CD39 and CD73 within the CD56^+^CD16^−^NK cell subset, which reached statistical significance in at least 2 of 4 tumor entities (Fig. 3F).

As with the CD8^+^ T cells of BM aspirates, NK cells also showed increased expression of at least one target molecule of the TIGIT/PVRIG pathway axis or the CD39/CD73 purinergic pathway in comparison with NMCs (BC: TIGIT, PVRIG, CD226; PC: PVRIG; NSCLC: TIGIT, CD39, CD73; MM: PVRIG, CD226, CD39). Again, there was no clear difference between solid tumor aspirates and those of MM patients. Overall, TIGIT and PVRIG were expressed mainly on CD56^−^CD16^+^ NK cells, while CD39 and CD73 were dominantly expressed by the CD56^+^CD16^−^ NK cell subset.

### M2-like macrophages in bone metastasis dominantly express TIGIT and PVRL4 or CD112 and CD155

Additionally, to T and NK cells, it was recently discovered, that macrophages also express ligands and receptors of co-inhibitory checkpoints including TIGIT and TIM-3 [43]. Therefore, we analyzed the expression of TIGIT and its ligands CD112, CD155 and PVRL4 on BM-derived macrophages. In addition, expression of the two ectonucleotidase CD39 and CD73 was analyzed on macrophages. As previously described MFC expression analyses were conducted on M0 (CD68^+^CD14^+^), M2-like (CD163^+^CD86^+^) and M1-like (CD163^−^CD86^+^) macrophages.

**Fig. 4:**
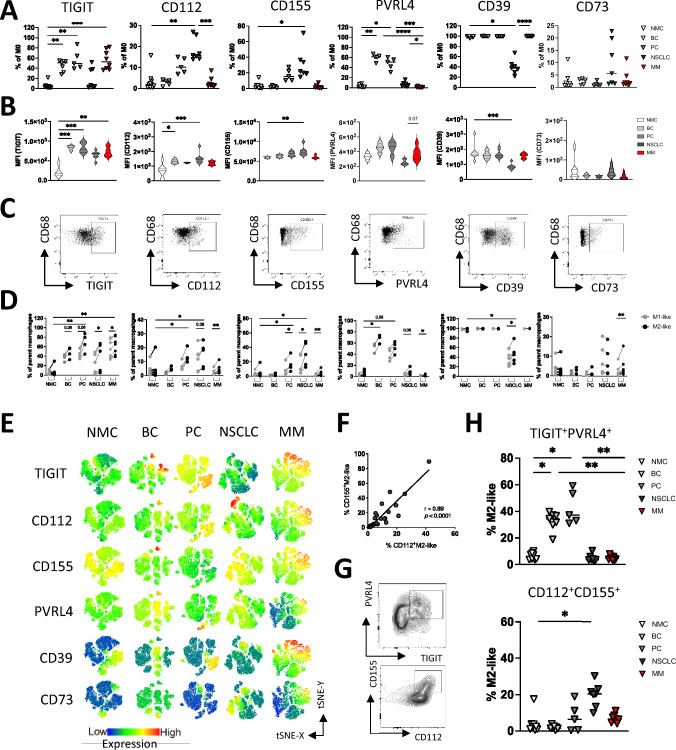
M2-like macrophages in bone metastasis dominantly express TIGIT and PVRL4 or CD112 and CD155. Expression of TIGIT, CD112, CD155, PVRL4, CD39 and CD73 on macrophages was assessed by multiparametric flow (MFC) analyses. The cohort included bone aspirates from patients with breast cancer (BC *n* = 6), patients with prostate cancer (PC *n* = 5), patients with non-small cell lung cancer (NSCLC *n* = 7), and patients with multiple myeloma (MM *n* = 10). MFC data were compared to age-matched mononuclear cells of bone aspirates from non-malignant patients undergoing spine surgery (NMC *n* = 10). **A** Frequencies of TIGIT, CD112, CD155, PVRL4, CD39 and CD73 expressing M0 macrophages. **B** Median fluorescence intensity (MFI) of the respective target proteins on indicated M0 macrophages. **C** Flow plots illustrate the target protein expression on M0 macrophages. **D** Expression of the target protein was compared between CD163^−^CD86^+^ M1-like versus CD163^+^CD86^+^ M2-like macrophages. **E** T-distributed stochastic neighbor embedding (tSNE) heat maps illustrate the expression and location of target proteins on M0 macrophages. **F** Correlation analysis of CD112 and CD155 on CD163^+^CD86^+^ M2-like macrophages. **G** Respective flow plots illustrate co-expression of TIGIT with PVRL4 and CD112 with CD155. **H** Summary data show the co-expression of the indicated target proteins on CD163^+^CD86^+^ M2-like macrophages. Frequencies are displayed with the medians. *p* Values were obtained by the ANOVA and Kruskal Wallis test. Pearson’s test was used to test for correlations. *p < 0.05, **p < 0.01, ***p < 0.001, ****p < 0.0001

Except the NSCLC aspirates, all BM aspirates showed an increased proportion of TIGIT^+^CD68^+^CD14^+^ macrophages compared to the NMC cohort. CD112^+^ and CD155^+^ macrophages were more frequently expressed in NSCLC aspirates, and with a trend also in PC aspirates, while the third TIGIT ligand, PVRL4, was in the majority expressed by macrophages from BC and PC aspirates. Regarding the purinergic system, CD39 was expressed in almost 100% of macrophages from the cohorts, except NSCLC aspirates. No relevant expression of CD73 could be detected on the macrophages (Fig. 4A). Interestingly, only the target receptor TIGIT was found to be aberrantly expressed by macrophages of aspirates from MM patients. The remaining markers were comparable to those in NMCs. The data of the frequency of positive cells, were confirmed by the results of the median fluorescence intensity for the respective markers (MFI; Fig. 4B). Expression of TIGIT, CD112, CD155, PVRL4, CD39 and CD73 are as illustrated for the sake of clarity (Fig. 4C).

Similar to the NK cell subsets, an increased expression of molecules of the TIGIT pathway axis was observed on M2-like macrophages. Significantly higher frequencies of TIGIT^+^, CD112^+^, CD155^+^, and PVRL4^+^ cells were observed within the M2-like macrophages versus M1-like macrophages, whereas expression of CD39 and CD73 were not restricted to M2-like or M1-like subsets (Fig. 4D).

In the tSNE analyses, TIGIT and PVRL4 as well as CD112 and CD155 were expressed in the same regions of M2-like macrophages (Fig. 4E). Correlation analyses revealed an association between CD112 and CD155 expression on M2-like macrophages (due to the small number of cases, the malignant cohorts were combined here; Fig. 4F). As exemplified by FACS plots on M2-like macrophages (Fig. 4G) co-expression analyses revealed that M2-like macrophages from patients with BC and PC more frequently co-expressed TIGIT and PVRL4, whereas macrophages from patients with NSCLC displayed higher co-expression of CD112 and CD155 compared with NMCs. (Fig. 4H).

In summary macrophages from BM of all 3 solid tumors and of the MM lesions were also found to express two target molecules of the TIGIT pathway more frequently in each tumor type than in NMC aspirates (BC: TIGIT, PVRL4; PC: TIGIT, PVRL4; NSCLC: CD112, CD155; MM: TIGIT, CD155). Interestingly, expression of the target molecules was restricted to the M2-like macrophages. In contrast to NK and T cells, no increased expression of CD39 and CD73 could be detected, nor any difference between M1 and M2-like with BM-derived macrophages*.*

### Blockade of TIGIT and CD39 augmented lysis of tumor cells

The presented immunophenotypic analyses revealed aberrant expression of the inhibitory receptor TIGIT and the ectoenzyme CD39 in several immune cell populations of BM. We have previously shown that both pathways synergistically can boost anti-tumor immunity in other tumor types [27]. Here, we analyzed the therapeutic potential of blocking TIGIT and CD39 on the cytotoxicity of peripheral blood mononuclear cells (PBMCs). The aim of the functional blockade was to achieve an increase in PBMC-mediated lysis of tumor cells. The functional investigations were carried out exemplarily in a myeloma model. For this purpose, two myeloma cell lines were used that expressed CD39 at different frequencies. Both cell lines were described to express the TIGIT ligands CD112 and CD155 [44].

**Fig. 5:**
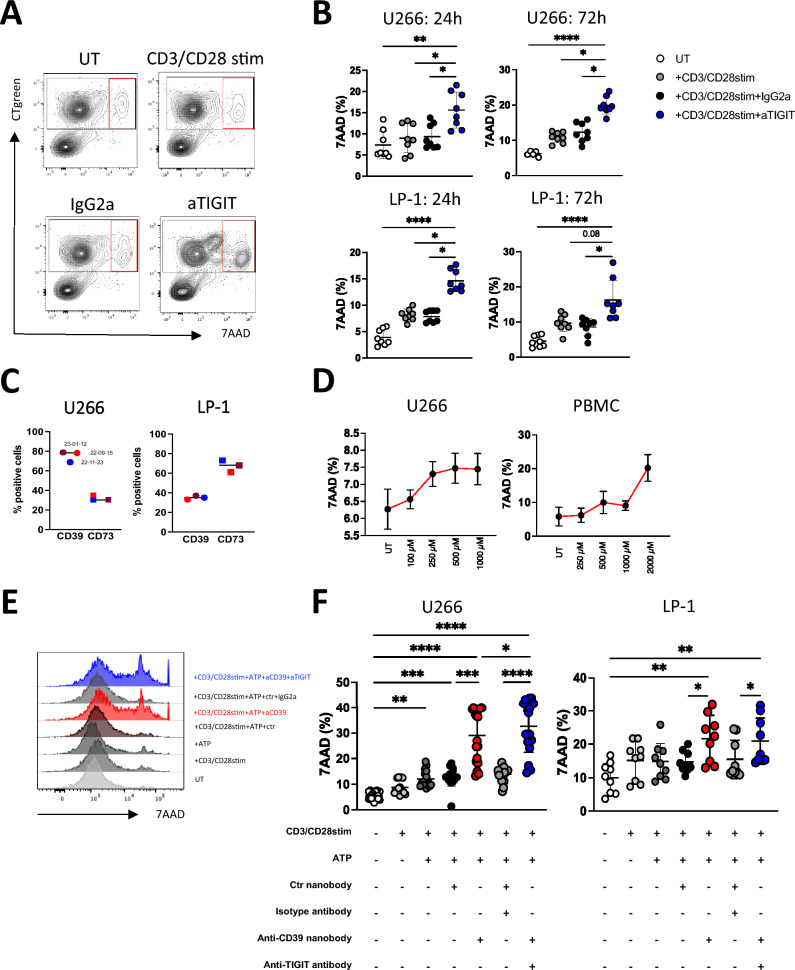
Blockade of TIGIT and CD39 augmented lysis of tumor cells. Blockade of TIGIT and CD39 was analyzed for peripheral blood mononuclear cells (PBMC) co-cultured with myeloma (MM) cells over 24 h and 72 h in vitro. **A** Representative flow cytometry plots illustrate the frequency of 7AAD^+^ tumor cells after treatment. **B** Effects of the single TIGIT blockade on CD3/CD28 stimulated PBMC-mediated lysis was analyzed by detecting the frequency of lysed U266 and LP-1 MM cells. Tumor cell lysis was defined by double positivity of CT green and 7-AAD measured by MFC analysis. Summary data are depicted as the mean ± SD fold changes, relative to the untreated and isotype treated condition for the MM cell lines U266 (*n* = 3), LP-1 (*n* = 3). **C** Representative frequencies showing expression status of CD39 and CD73 on U266 and LP-1 MM cells at different time points. **D** Cytotoxic effects of exogenous adenosine triphosphate (ATP) were analyzed in vitro for U266 MM cells and PBMCs testing ATP concentrations from 0 to 2000 µM. Cytotoxicity was analyzed by measuring the frequency of lysed (7-AAD^+^) cells (*n* = 3). **E** Exemplary FACS histograms demonstrating 7AAD expression under respective treatment strategies. **F** Blockade of CD39 and TIGIT under influence of exogenous ATP on CD3/CD28 stimulated PBMC was analyzed regarding PBMC-mediated killing of U266 cells (*n* = 5) and LP-1 cells (*n* = 4). All measurements were performed in technical triplicates. *p* values were obtained by the Wilcoxon test. * *p* < 0.05, ** *p* < 0.01, *** *p* < 0.001

As exemplary shown in Fig. 5A, MFC analyses revealed in both myeloma cell lines tested, single blockade of the TIGIT receptor resulted in enhanced CD3/CD28 stimulated PBMC cytotoxicity. TIGIT blockade was able to increase PBMC-mediated lysis of U266 and LP-1 cells at 24 h and 72 h (Fig. 5B). As CD39 was expressed by the MM cell lines U266 and LP-1 (Fig. 5C) the functional blockade of the ectoenzyme CD39 was conducted by testing a newly developed nanobody construct [38]. The functional evaluation of the ectonucleotidase CD39 requires the enzyme’s substrate, exogenous adenosine triphosphate (ATP). In a preliminary experiment we tested which concentrations of exogenous ATP itself have a cytotoxic effect on the included cell populations. In vitro ATP-mediated cytotoxicity on U266 MM cells and PBMCs was analyzed using ATP concentrations ranging from 0 to 2000 µM. The study revealed for both, the target and the effector population, that ATP concentrations up to 1000 µM resulted in cytotoxicity rates below 10% (7AAD ^+^ cells; Fig. 5D). Regarding PBMC-mediated lysis of tumor cells, the subsequent cytotoxicity assays showed that the blockade of CD39 led to an increased CD3/CD28 stimulated PBMC-mediated lysis of MM cells (Fig. 5E, F). The effects were more enhanced in the U266 myeloma cell line, this might be due to a more frequent CD39 expression. Due to these promising results, we tested the effects of the additional TIGIT blockade with an inhibition of the purinergic pathway on PBMC-mediated killing. Dual blockade of CD39 together with TIGIT significantly increased MM cell lysis in one of two cell lines in comparison with a single blockade or in both cell lines in comparison with the control treatment (Fig. 5E, F).

In summary, the combined blockade achieved significant lysis of tumor cells. Our investigations showed that lysis was higher in tumor cells with a higher CD39 expression than in cell lines with a lower expression status.

## Discussion

The present study detected a universal shift of immune cell subsets in aspirates of BM compared to that seen in NMC aspirates. The fraction of CD8^+^ T cells was lower, while the infiltration rate of immunosuppressive CD56^+^CD16^−^ NK cells and CD163^+^CD86^+^ M2-like macrophages were significantly enriched in all 4 tumor entities.

Analyzing novel immunological target molecules of the TIGIT axis and the purinergic pathway, an increased frequency of CD8^+^ T cells co-expressing TIGIT with PVRIG or CD39 was characteristic for all BM aspirates, in contrast to NMC. Similarly, BM-derived NK cells more frequently co-expressed TIGIT and PVRIG within the cytotoxic CD56^−^CD16^+^ NK cells. In contrast co-expression of CD39 and CD73 was found within the regulatory CD56^+^CD16^−^ subset. Additionally, BM-derived M2-like macrophages either co-expressed TIGIT with PVRL4 or CD112 with CD155.

Based on the results that TIGIT and CD39 were aberrantly expressed in almost all analyzed cell subsets in BM, further functional investigation of the two target molecules was carried out.

Using a myeloma model, our in vitro studies showed that a (combined) blockade of TIGIT and CD39 leads to increased lysis of tumor cells.

Bone represents a common site for metastasis in solid tumors, including BC, PC, and lung cancer which emphasizes the importance of this location of metastasis [45]. Factors that favor BM occurrence might include the high blood flow in the bone marrow, providing a fertile soil for circulating tumor cells, but also the immunosuppressive bone microenvironment, crucial for maintaining the hematopoietic stem cell niche [46, 47]. In addition to these unfavorable inflammatory factors, the present study revealed significant differences in the composition of the immune cell repertoire between BM and NMC aspirates. The study showed that there were entity-specific differences in the infiltration rate of total CD3^+^ T-, NK cells and macrophages. Moreover, also uniform changes occurred in all 4 tumor entities. The CD8^+^ T cells fraction was uniformly reduced whereas CD56^+^CD16^−^ NK cells and M2-like macrophages were enriched in BM aspirates. This immunological shift has already been described as prognostically unfavorable in primary tumor tissues and as one of the causes of poor responsiveness to classic ICIs [48]. Our findings align with recent research indicating that the CD4/CD8 ratio plays a crucial role in solid cancer patients undergoing ICI therapy, where a reduced CD8^+^ T cell population correlates with decreased PFS and OS [49, 50]. Also, presence of CD56^+^CD16^−^ NK and M2-like macrophages in primary tumor sites or circulating in the peripheral blood have been recently identified as crucial for development of metastasis, invasion, and treatment resistance [51, 52]. Surprisingly, the study showed no substantial differences when comparing immune cell infiltration rates between solid tumor entities and MM aspirates. To our knowledge, there are no phenotypic studies on bone metastases with which the results can be further discussed. The characteristic immune cell distribution in BM could indicate that the cytotoxic immune cell populations may exhibit a weakened cytotoxic response due to the interactions of inhibitory coreceptors. Regarding the regulatory cell subsets, one possibility to exert their immunosuppressive effect might be the generation of immunosuppressive adenosine via the ectoenzymes CD39 and CD73.

BM represent a negative prognostic factor in advanced cancer patients undergoing ICI therapy and seem to exhibit inferior clinical responses compared to metastatic lesions in other sites of disease [53, 54]. To increase anti-tumor cytotoxicity identification of novel immune checkpoint molecules is necessary. In the present study TIGIT was co-expressed with PVRIG and CD39 by CD8^+^ T cells in BM. In line with our data positive (co-) expression of TIGIT and PVRIG or CD39 was recently detected on CD8^+^ T cells derived from the primary site of several tumor types including lung, breast cancers and acute leukemia [27, 55, 56]. In first preclinical models, the interaction of TIGIT and/or PVRIG with their ligands has been demonstrated to be associated with reduced CD8^+^ T cell proliferation, activation and T cell cytokine production [55, 57]. Blockade of TIGIT ± CD39 blockade was able to restore effector function of CD8^+^ T cells and NK cells and significantly increased lysis of tumor cells [58]. Nevertheless, the blockade of TIGIT has not yet been convincing in the ongoing clinical studies [59]. Even if the effects by a TIGIT blockade are promising further synergistic immunologic pathways need to be identified to establish multi-specific targeting strategies to enhance and stabilize anti-tumor immunity.

Notably, we could not detect differences of the selected checkpoints on CD4^+^ T cells from BM. However, we were not able to further differentiate into conventional and regulatory CD4^+^ T cells. Recently, increased expression of TIGIT has been shown on regulatory CD4^+^ T cells of primary tumor tissue as well as during chronic infections [60, 61]. The lack of CD4 differentiation represents a limitation of our study.

As immunotherapeutic approaches are increasingly focusing on the reactivation of NK cells, the present study subsequently analyzed the expression status of the TIGIT axis and purinergic pathway in this population [16]. A characteristic co-expression of TIGIT and PVRIG on CD56^−^CD16^+^ NK cells was found. In line with our data, high expression of TIGIT and PVRIG on cytotoxic NK cells has been recently described as sign of exhaustion in NK cells infiltrating primary tumor sites [62]. Regarding the characteristic co-expression of CD39 and CD73 on regulatory CD56^+^CD16^−^ NK cells, we and others recently found in line with our data, that circulating or tissue-derived CD56^+^ NK cells from cancer patients highly express CD39 and CD73 and thereby acting immunosuppressive via extracellular adenosine generation [27]. Moreover, first functional evaluation proofed that blockade of TIGIT and/or PVRIG prevented NK cell exhaustion and promotes NK cell–dependent tumor immunity in solid and hematological tumor models [24, 63]. In addition, the combined blockade of the TIGIT axis together with the purinergic pathway was superior to a single blockade in preclinical models as well as in the present study [27].

Across all investigated tumor entities, M2-like macrophages were more frequently found in BM aspirates than in NMC aspirates. CD163 was used as M2-like marker based on previous analyses of bone marrow derived macrophages. As recently showed CD204 and CD206, two further M2-like associated markers were in the majority co-expressed with CD163. Additionally, no changes in the expression of co-regulatory receptors including TIGIT on CD204^+^ or CD206^+^ M2 macrophages compared to CD163^+^ macrophages were found. For simplicity, we restricted the phenotyping on CD163, CD86, CD68, and CD14 [26, 64, 65]. It was recently demonstrated that disseminated cancer cells within the bone marrow can be suppressed by BM-resident macrophages and cytotoxic T cells which form a crucial line of defense against cancer cells [16]. These data once again emphasize the relevance of macrophages in immune oncology. In line with the metastatic situation, we and others recently detected expression of co-regulatory molecules including TIGIT on M2-like macrophages derived from tumor tissue (24). The impact of TIGIT expressed by macrophages has recently been shown by Chen et al. demonstrating in a mouse model that TIGIT/PVR signaling suppressed xenogeneic M1 polarization and c-Maf-regulated proinflammatory cytokine production [66]. Moreover, the present study revealed that the PVR‐like family members CD112, and CD155 are expressed by BM-derived macrophages. While expression of CD112 has not previously been described on macrophages, in tumor-associated macrophages of colorectal carcinoma a higher expression of CD155 was detected than in macrophages from para-tumor or adjacent normal tissue [67]. Expression of CD155 was associated with an M2-like phenotype and a higher expression of interleukin (IL)-10 and transforming growth factor (TGF)-β, as well as with colorectal tumor stages III/IV and with a shorter survival [67].

While PVRL4 has previously been reported to be overexpressed on tumor cells, this study demonstrates its expression on macrophages as a TIGIT ligand.

The functional investigation of the blockade of TIGIT and CD39 was conducted in allogeneic immune cell-based assays in vitro [27]. The cytotoxic assays showed an increased PBMC-mediated lysis of myeloma cells using a single blockade or combining blockade of TIGIT and CD39. The synergistic effects were at least evident in one cell line (U266). PBMCs were applied as they contain different leukocyte populations thereby better reflecting the immunological cell diversity than a single isolated effector cell population. There are also functional data on monoclonal antibodies against CD39. However, the use of nanobody constructs allows better access to the catalytic center of CD39 as well as the advantage of easily engineering bispecific formats [38]. We are currently working on their in vivo implementation. For the anti-TIGIT antibody (A15153G), we were able to show in previous studies that its use leads to increased T-cell and NK cell-mediated lysis of acute myeloid leukemia (AML) cells or breast cancer cells [27, 68].

Our study presents a comprehensive phenotypic overview on different BM-derived immune cell populations. The study focused primarily on the expression of new clinically useful checkpoint molecules. However, this study has some shortcomings that are common to translational cohort studies. The number of analyzed cells and the number of antibodies that could be stained in a single FACS panel was limited. Even though the donors did not differ in age, the patient groups for the individual tumor entities were quite small. Also, because subsequent treatment took place either in a different center and or no additional biopsy was indicated, no material from the primary tumor was available for direct comparison with the bone metastases.

In conclusion, the shifted distribution of immune cell subsets and aberrant expression of several molecules from the TIGIT axis and purinergic pathways underscore their relevance for immune cell interactions in BM. Both the TIGIT axis and the purinergic pathway were found to be relevant in all 4 tumor entities, albeit with expression differences. Blockade of CD39 and TIGIT could represent an interesting approach to increase anti-tumor immunity in BM.

## Supplementary Information

Below is the link to the electronic supplementary material.

Supplementary file1 (PPTX 7082 kb)

## Data Availability

The datasets used and/or analyzed during the current study are available from the corresponding authors on reasonable request (f.brauneck@uke.de).

## Abbreviations

CD39: Ectonucleoside triphosphate diphosphohydrolase 1
CD73: Ecto-5′-nucleotidase
CD111: PVRL1
CD112: PVRL2
CD155: PVR
CD163: Haptoglobin–hemoglobin scavenger receptor
CD226: Cluster of Differentiation 226/DNAM
FMO: Fluorescence minus one
IFN-γ: Interferon-γ
MFC: Multiparametric flow cytometry
MFI: Mean fluorescence intensity
NMC: Non-malignant control group
OS: Overall survival
PB: Peripheral blood
PVRIG: Poliovirus receptor-related immunoglobulin domain-containing
PVRL1: Poliovirus receptor-related 1
PVRL2: Poliovirus receptor-related 2
PVRL4: Poliovirus receptor-related 4
TAMs: Tumor-associated macrophages
TIGIT: T cell immunoreceptor with Ig and ITIM domains
TME: Tumor microenvironment
TNF: Tumor necrosis factor
tSNE: T-distributed stochastic neighbor embedding
