# Supplementary material for: Expression of the TIGIT axis and the CD39/CD73 purinergic pathway in bone metastasis-derived immune cells
Source: Cancer Immunol Immunother. 2025 Apr 24;74(6):182. doi: 10.1007/s00262-025-04030-2 (PMC12022200; doi:10.1007/s00262-025-04030-2)
Supplement: Supplementary file 1 — Supplementary file1 (PPTX 7082 kb) [file 262_2025_4030_MOESM1_ESM.pptx]

## Slide 1
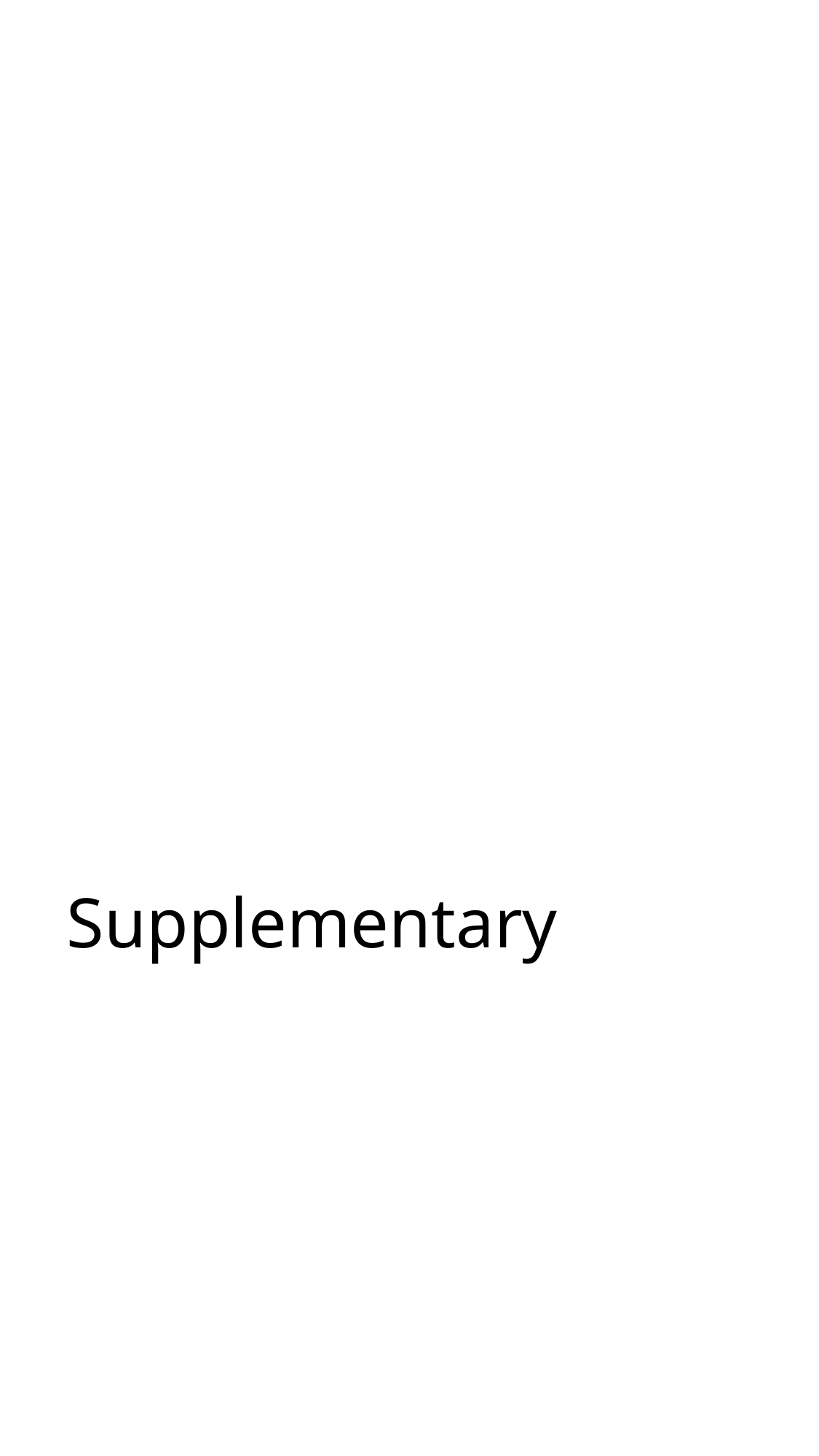

# Supplementary

## Slide 2
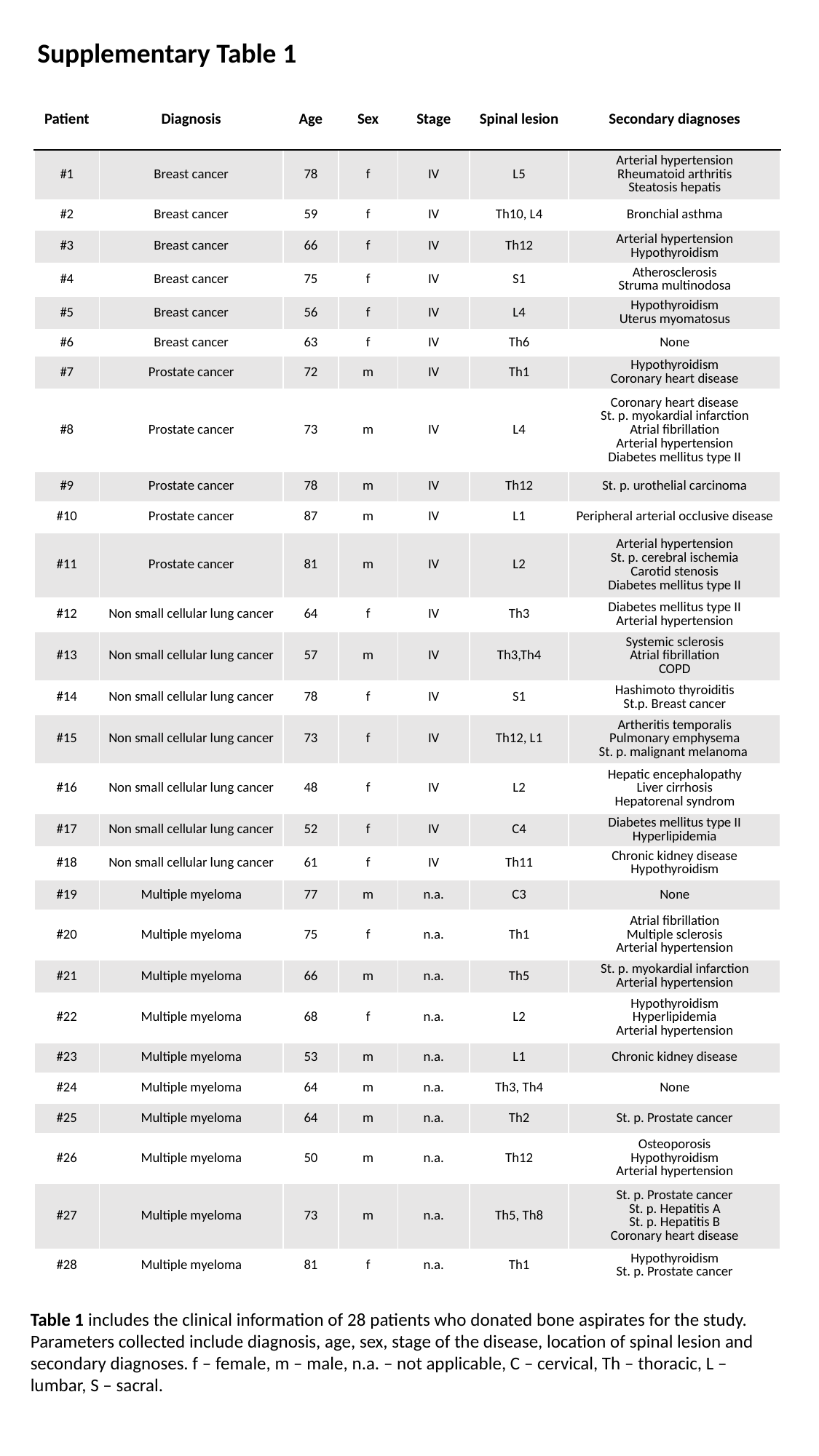

Supplementary Table 1
| Patient | Diagnosis | Age | Sex | Stage | Spinal lesion | Secondary diagnoses |
| --- | --- | --- | --- | --- | --- | --- |
| #1 | Breast cancer | 78 | f | IV | L5 | Arterial hypertension Rheumatoid arthritis Steatosis hepatis |
| #2 | Breast cancer | 59 | f | IV | Th10, L4 | Bronchial asthma |
| #3 | Breast cancer | 66 | f | IV | Th12 | Arterial hypertension Hypothyroidism |
| #4 | Breast cancer | 75 | f | IV | S1 | Atherosclerosis Struma multinodosa |
| #5 | Breast cancer | 56 | f | IV | L4 | Hypothyroidism Uterus myomatosus |
| #6 | Breast cancer | 63 | f | IV | Th6 | None |
| #7 | Prostate cancer | 72 | m | IV | Th1 | Hypothyroidism Coronary heart disease |
| #8 | Prostate cancer | 73 | m | IV | L4 | Coronary heart disease St. p. myokardial infarction Atrial fibrillation Arterial hypertension Diabetes mellitus type II |
| #9 | Prostate cancer | 78 | m | IV | Th12 | St. p. urothelial carcinoma |
| #10 | Prostate cancer | 87 | m | IV | L1 | Peripheral arterial occlusive disease |
| #11 | Prostate cancer | 81 | m | IV | L2 | Arterial hypertension St. p. cerebral ischemia Carotid stenosis Diabetes mellitus type II |
| #12 | Non small cellular lung cancer | 64 | f | IV | Th3 | Diabetes mellitus type II Arterial hypertension |
| #13 | Non small cellular lung cancer | 57 | m | IV | Th3,Th4 | Systemic sclerosis Atrial fibrillation COPD |
| #14 | Non small cellular lung cancer | 78 | f | IV | S1 | Hashimoto thyroiditis St.p. Breast cancer |
| #15 | Non small cellular lung cancer | 73 | f | IV | Th12, L1 | Artheritis temporalis Pulmonary emphysema St. p. malignant melanoma |
| #16 | Non small cellular lung cancer | 48 | f | IV | L2 | Hepatic encephalopathy Liver cirrhosis Hepatorenal syndrom |
| #17 | Non small cellular lung cancer | 52 | f | IV | C4 | Diabetes mellitus type II Hyperlipidemia |
| #18 | Non small cellular lung cancer | 61 | f | IV | Th11 | Chronic kidney disease Hypothyroidism |
| #19 | Multiple myeloma | 77 | m | n.a. | C3 | None |
| #20 | Multiple myeloma | 75 | f | n.a. | Th1 | Atrial fibrillation Multiple sclerosis Arterial hypertension |
| #21 | Multiple myeloma | 66 | m | n.a. | Th5 | St. p. myokardial infarction Arterial hypertension |
| #22 | Multiple myeloma | 68 | f | n.a. | L2 | Hypothyroidism Hyperlipidemia Arterial hypertension |
| #23 | Multiple myeloma | 53 | m | n.a. | L1 | Chronic kidney disease |
| #24 | Multiple myeloma | 64 | m | n.a. | Th3, Th4 | None |
| #25 | Multiple myeloma | 64 | m | n.a. | Th2 | St. p. Prostate cancer |
| #26 | Multiple myeloma | 50 | m | n.a. | Th12 | Osteoporosis Hypothyroidism Arterial hypertension |
| #27 | Multiple myeloma | 73 | m | n.a. | Th5, Th8 | St. p. Prostate cancer St. p. Hepatitis A St. p. Hepatitis B Coronary heart disease |
| #28 | Multiple myeloma | 81 | f | n.a. | Th1 | Hypothyroidism St. p. Prostate cancer |
Table 1 includes the clinical information of 28 patients who donated bone aspirates for the study. Parameters collected include diagnosis, age, sex, stage of the disease, location of spinal lesion and secondary diagnoses. f – female, m – male, n.a. – not applicable, C – cervical, Th – thoracic, L – lumbar, S – sacral.

## Slide 3
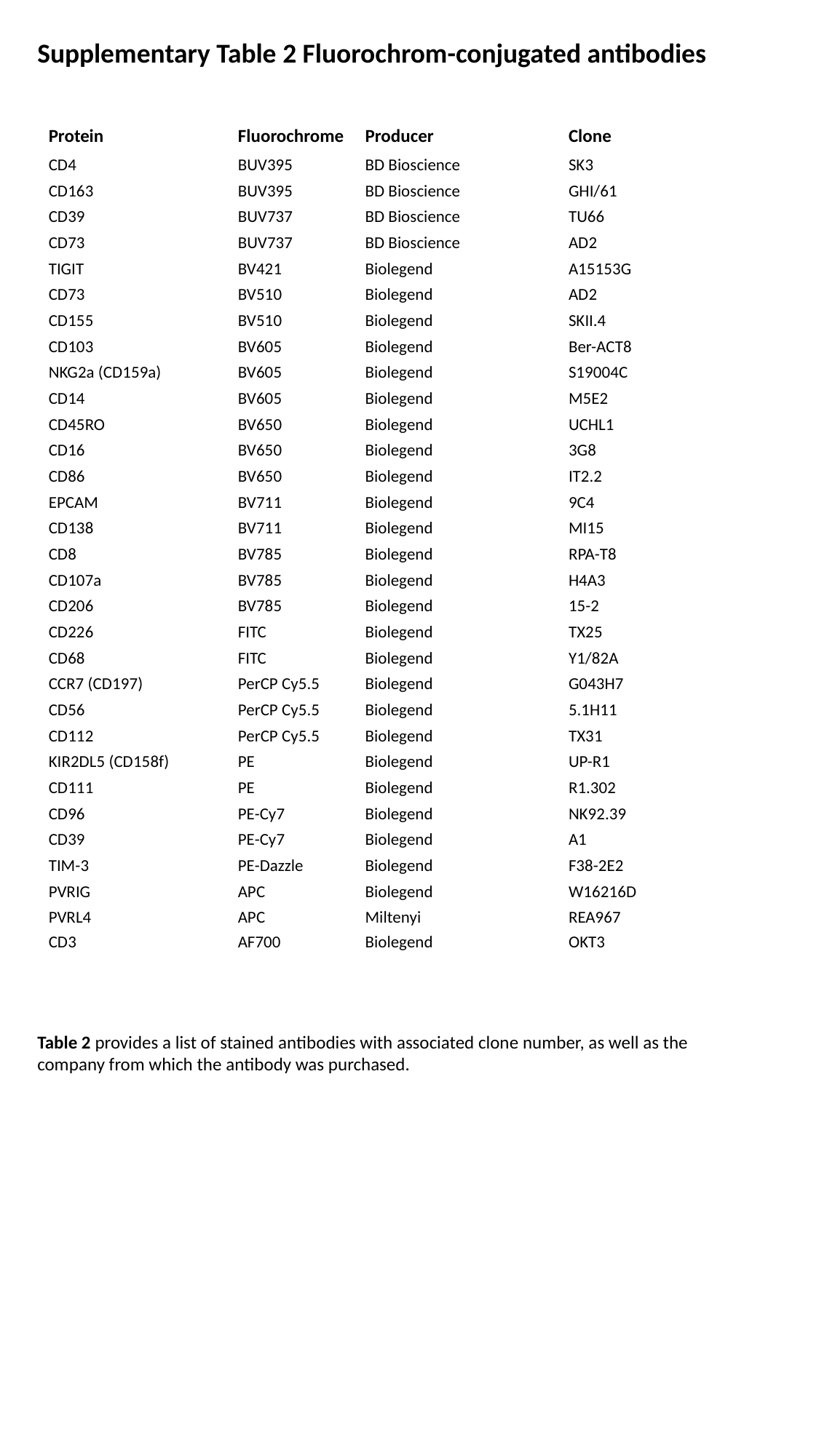

Supplementary Table 2 Fluorochrom-conjugated antibodies
| Protein | Fluorochrome | Producer | Clone |
| --- | --- | --- | --- |
| CD4 | BUV395 | BD Bioscience | SK3 |
| CD163 | BUV395 | BD Bioscience | GHI/61 |
| CD39 | BUV737 | BD Bioscience | TU66 |
| CD73 | BUV737 | BD Bioscience | AD2 |
| TIGIT | BV421 | Biolegend | A15153G |
| CD73 | BV510 | Biolegend | AD2 |
| CD155 | BV510 | Biolegend | SKII.4 |
| CD103 | BV605 | Biolegend | Ber-ACT8 |
| NKG2a (CD159a) | BV605 | Biolegend | S19004C |
| CD14 | BV605 | Biolegend | M5E2 |
| CD45RO | BV650 | Biolegend | UCHL1 |
| CD16 | BV650 | Biolegend | 3G8 |
| CD86 | BV650 | Biolegend | IT2.2 |
| EPCAM | BV711 | Biolegend | 9C4 |
| CD138 | BV711 | Biolegend | MI15 |
| CD8 | BV785 | Biolegend | RPA-T8 |
| CD107a | BV785 | Biolegend | H4A3 |
| CD206 | BV785 | Biolegend | 15-2 |
| CD226 | FITC | Biolegend | TX25 |
| CD68 | FITC | Biolegend | Y1/82A |
| CCR7 (CD197) | PerCP Cy5.5 | Biolegend | G043H7 |
| CD56 | PerCP Cy5.5 | Biolegend | 5.1H11 |
| CD112 | PerCP Cy5.5 | Biolegend | TX31 |
| KIR2DL5 (CD158f) | PE | Biolegend | UP-R1 |
| CD111 | PE | Biolegend | R1.302 |
| CD96 | PE-Cy7 | Biolegend | NK92.39 |
| CD39 | PE-Cy7 | Biolegend | A1 |
| TIM-3 | PE-Dazzle | Biolegend | F38-2E2 |
| PVRIG | APC | Biolegend | W16216D |
| PVRL4 | APC | Miltenyi | REA967 |
| CD3 | AF700 | Biolegend | OKT3 |
Table 2 provides a list of stained antibodies with associated clone number, as well as the company from which the antibody was purchased.

## Slide 4
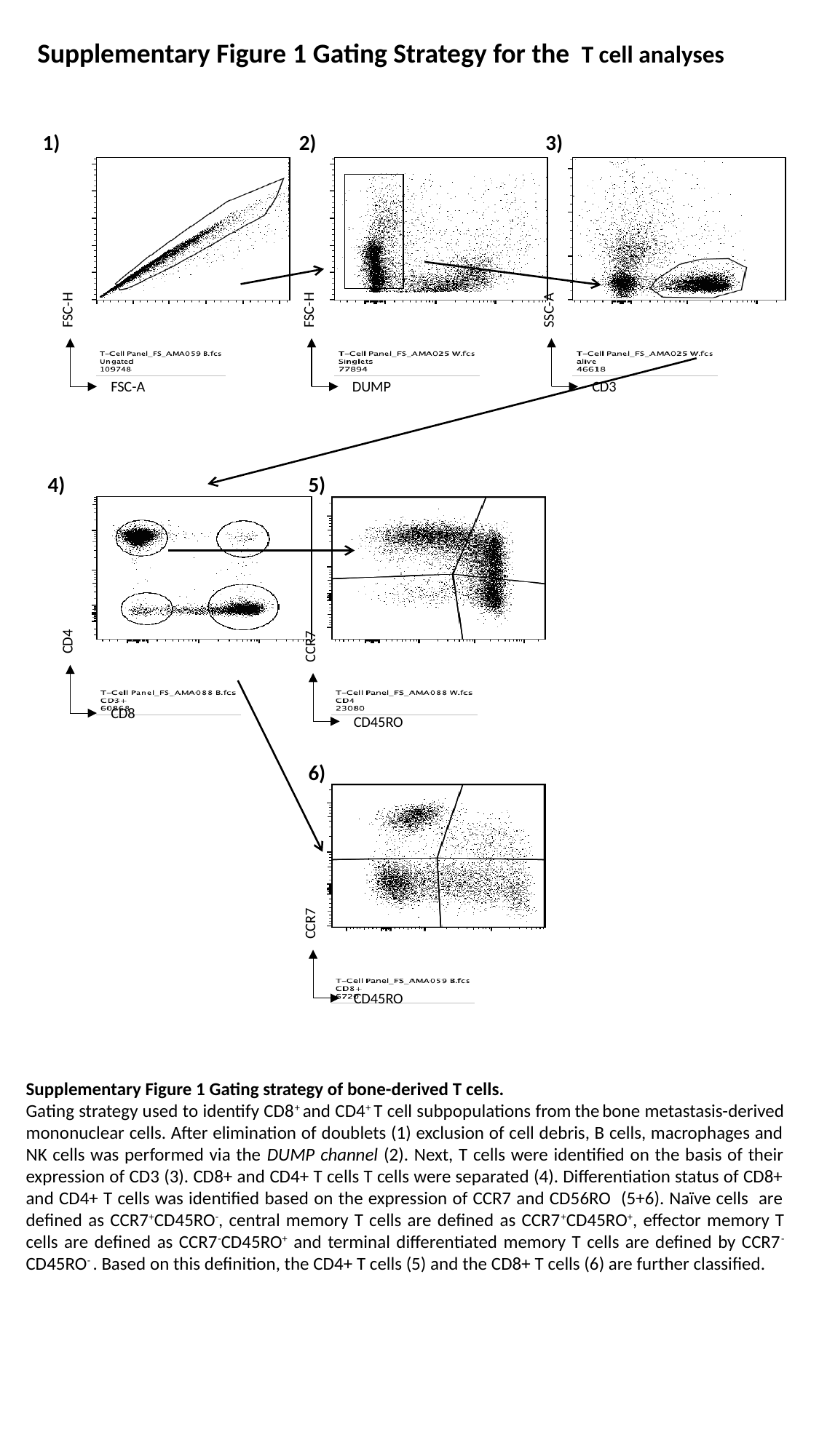

Supplementary Figure 1 Gating Strategy for the T cell analyses
1)
2)
3)
SSC-A
CD3
FSC-H
FSC-A
FSC-H
DUMP
4)
5)
CD4
CD8
CCR7
CD45RO
6)
CCR7
CD45RO
Supplementary Figure 1 Gating strategy of bone-derived T cells.
Gating strategy used to identify CD8+ and CD4+ T cell subpopulations from the bone metastasis-derived mononuclear cells. After elimination of doublets (1) exclusion of cell debris, B cells, macrophages and NK cells was performed via the DUMP channel (2). Next, T cells were identified on the basis of their expression of CD3 (3). CD8+ and CD4+ T cells T cells were separated (4). Differentiation status of CD8+ and CD4+ T cells was identified based on the expression of CCR7 and CD56RO (5+6). Naïve cells are defined as CCR7+CD45RO-, central memory T cells are defined as CCR7+CD45RO+, effector memory T cells are defined as CCR7-CD45RO+ and terminal differentiated memory T cells are defined by CCR7-CD45RO- . Based on this definition, the CD4+ T cells (5) and the CD8+ T cells (6) are further classified.

## Slide 5
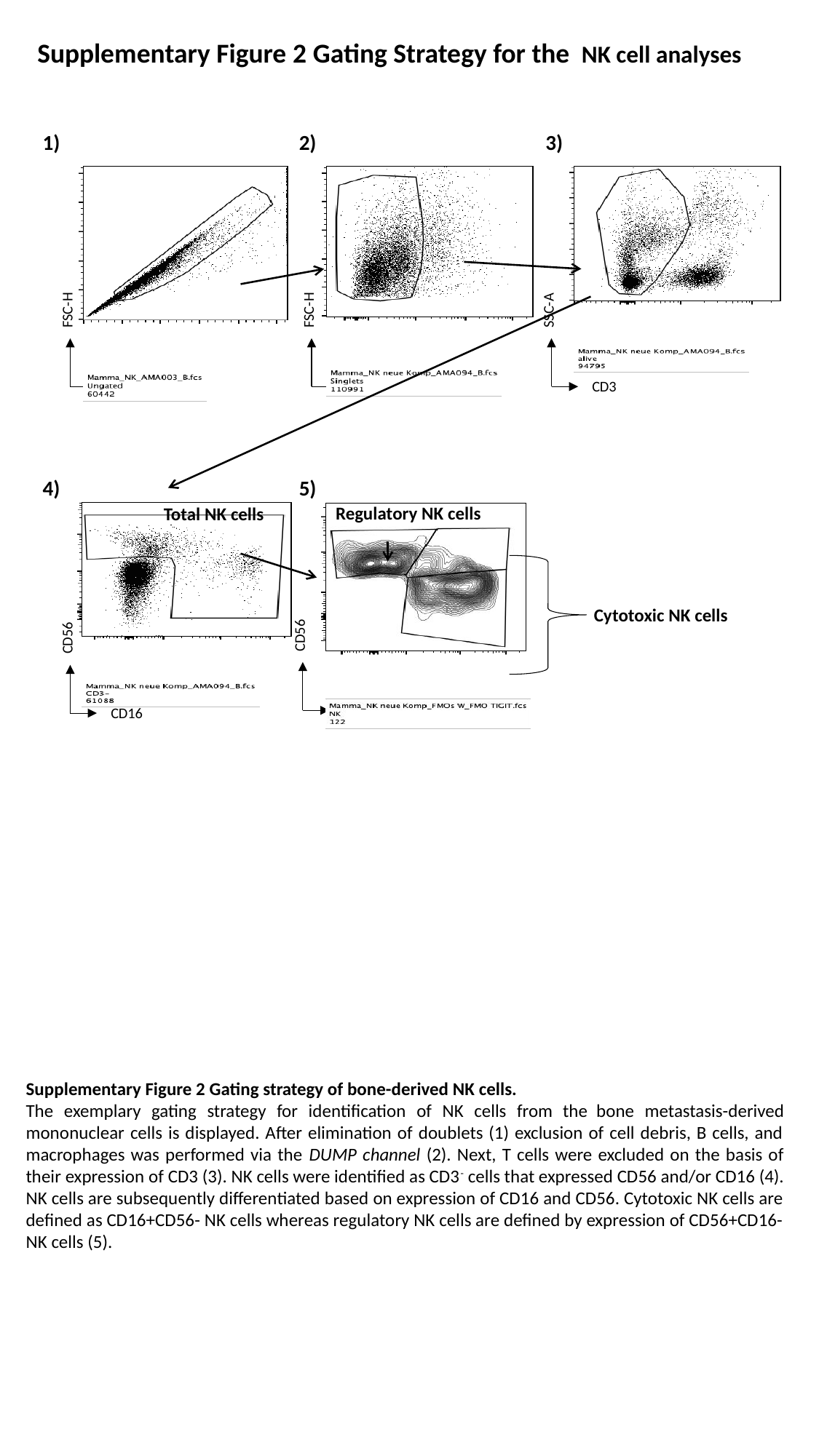

Supplementary Figure 2 Gating Strategy for the NK cell analyses
1)
2)
3)
SSC-A
CD3
FSC-H
FSC-A
FSC-H
DUMP
4)
5)
Regulatory NK cells
Total NK cells
CD56
CD16
CD56
CD16
Cytotoxic NK cells
Supplementary Figure 2 Gating strategy of bone-derived NK cells.
The exemplary gating strategy for identification of NK cells from the bone metastasis-derived mononuclear cells is displayed. After elimination of doublets (1) exclusion of cell debris, B cells, and macrophages was performed via the DUMP channel (2). Next, T cells were excluded on the basis of their expression of CD3 (3). NK cells were identified as CD3- cells that expressed CD56 and/or CD16 (4). NK cells are subsequently differentiated based on expression of CD16 and CD56. Cytotoxic NK cells are defined as CD16+CD56- NK cells whereas regulatory NK cells are defined by expression of CD56+CD16- NK cells (5).

## Slide 6
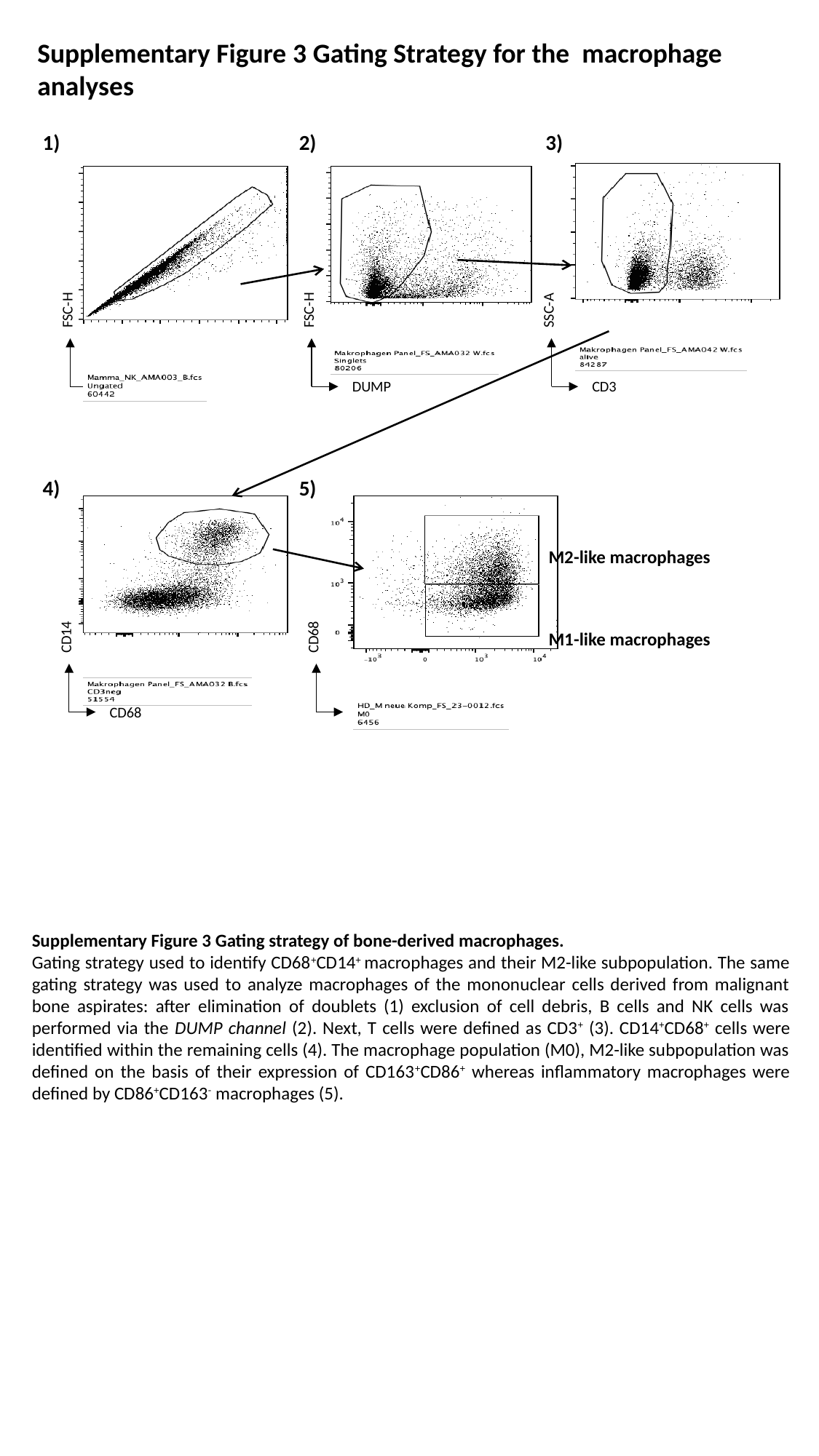

Supplementary Figure 3 Gating Strategy for the macrophage analyses
1)
2)
3)
SSC-A
CD3
FSC-H
FSC-A
FSC-H
DUMP
4)
5)
M2-like macrophages
CD14
CD68
CD68
CD163
M1-like macrophages
Supplementary Figure 3 Gating strategy of bone-derived macrophages.
Gating strategy used to identify CD68+CD14+ macrophages and their M2-like subpopulation. The same gating strategy was used to analyze macrophages of the mononuclear cells derived from malignant bone aspirates: after elimination of doublets (1) exclusion of cell debris, B cells and NK cells was performed via the DUMP channel (2). Next, T cells were defined as CD3+ (3). CD14+CD68+ cells were identified within the remaining cells (4). The macrophage population (M0), M2-like subpopulation was defined on the basis of their expression of CD163+CD86+ whereas inflammatory macrophages were defined by CD86+CD163- macrophages (5).

## Slide 7
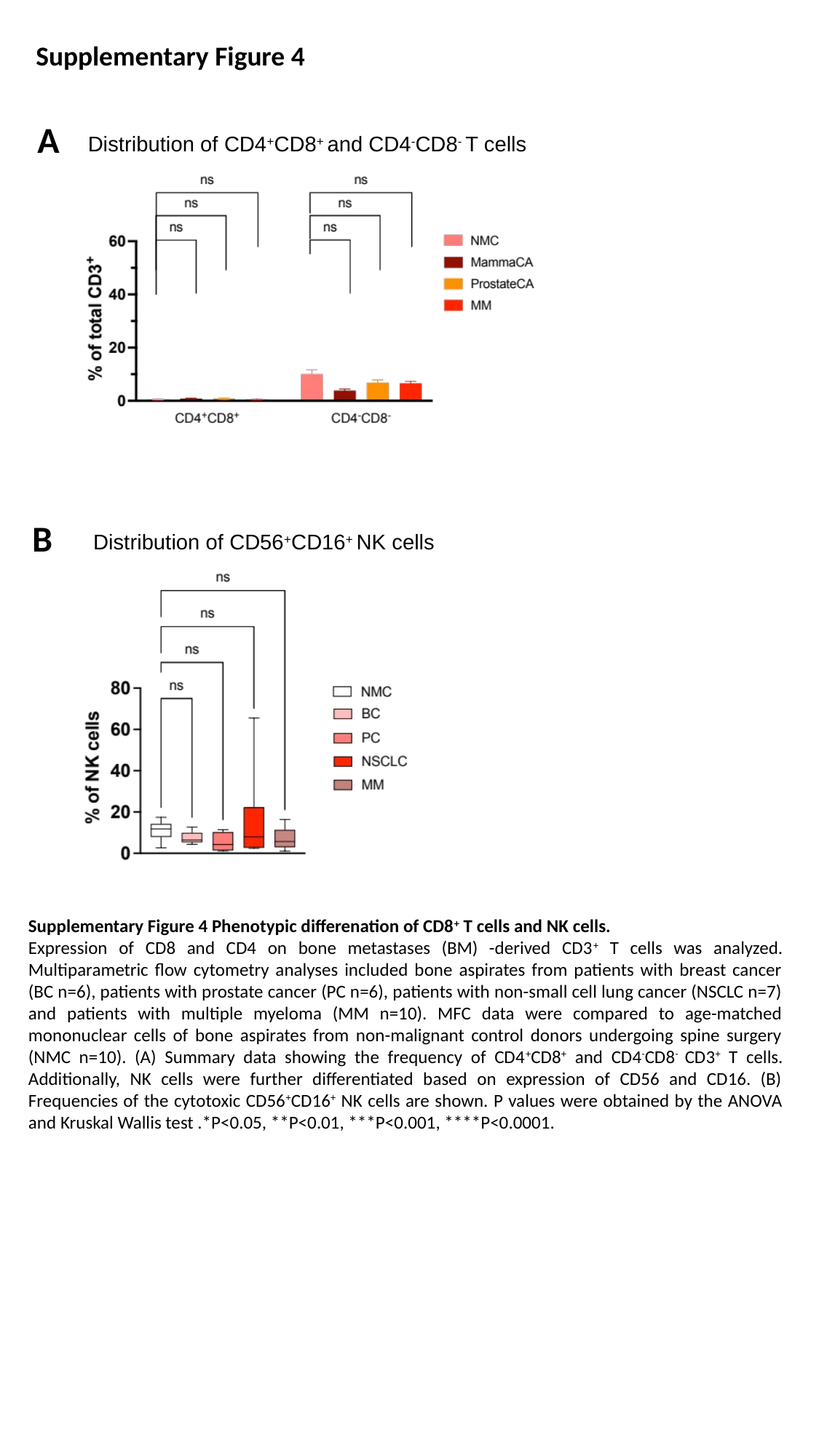

Supplementary Figure 4
A
Distribution of CD4+CD8+ and CD4-CD8- T cells
B
Distribution of CD56+CD16+ NK cells
Supplementary Figure 4 Phenotypic differenation of CD8+ T cells and NK cells.
Expression of CD8 and CD4 on bone metastases (BM) -derived CD3+ T cells was analyzed. Multiparametric flow cytometry analyses included bone aspirates from patients with breast cancer (BC n=6), patients with prostate cancer (PC n=6), patients with non-small cell lung cancer (NSCLC n=7) and patients with multiple myeloma (MM n=10). MFC data were compared to age-matched mononuclear cells of bone aspirates from non-malignant control donors undergoing spine surgery (NMC n=10). (A) Summary data showing the frequency of CD4+CD8+ and CD4-CD8- CD3+ T cells. Additionally, NK cells were further differentiated based on expression of CD56 and CD16. (B) Frequencies of the cytotoxic CD56+CD16+ NK cells are shown. P values were obtained by the ANOVA and Kruskal Wallis test .*P<0.05, **P<0.01, ***P<0.001, ****P<0.0001.

## Slide 8
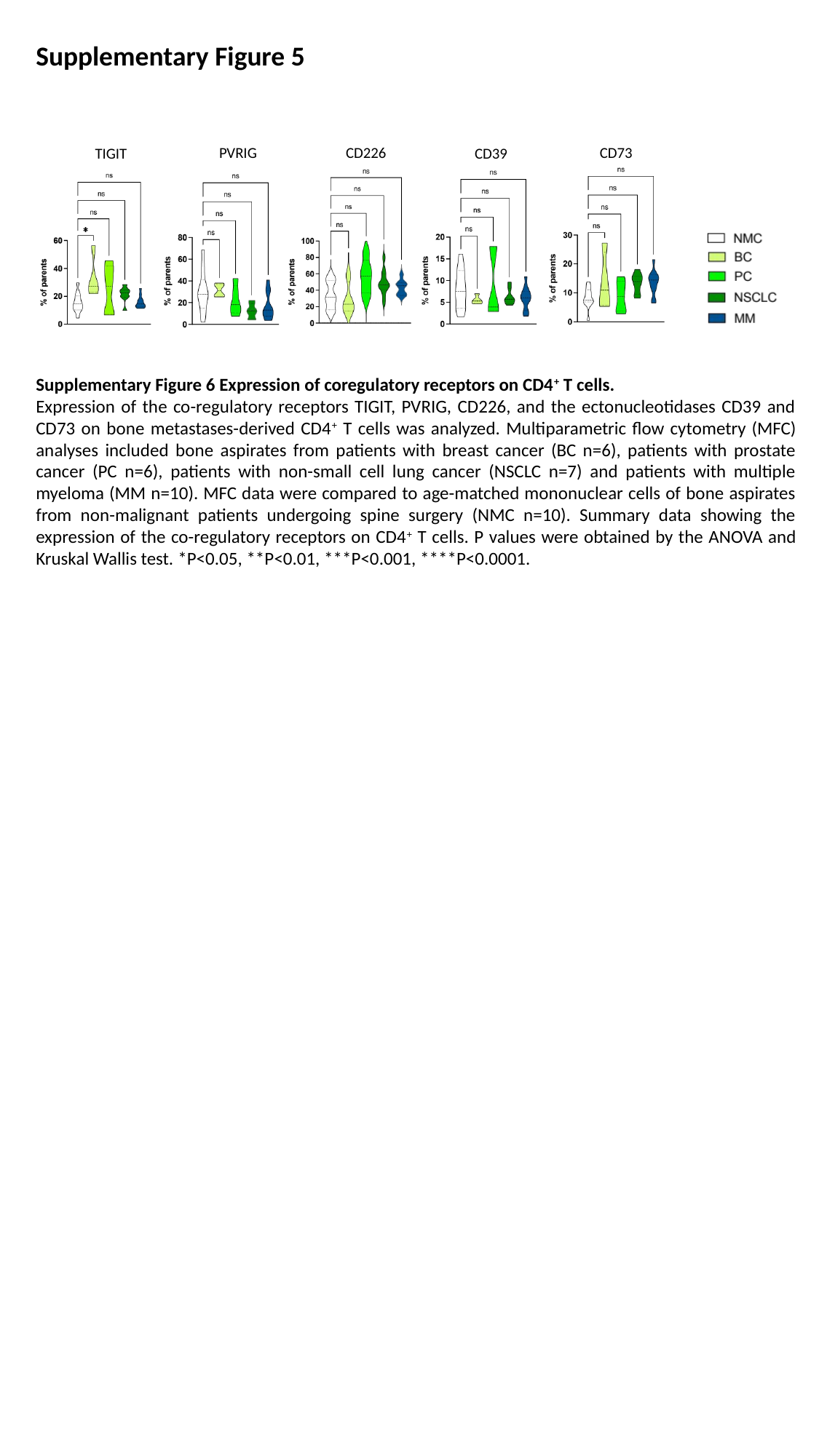

Supplementary Figure 5
PVRIG
CD226
CD73
CD39
TIGIT
Supplementary Figure 6 Expression of coregulatory receptors on CD4+ T cells.
Expression of the co-regulatory receptors TIGIT, PVRIG, CD226, and the ectonucleotidases CD39 and CD73 on bone metastases-derived CD4+ T cells was analyzed. Multiparametric flow cytometry (MFC) analyses included bone aspirates from patients with breast cancer (BC n=6), patients with prostate cancer (PC n=6), patients with non-small cell lung cancer (NSCLC n=7) and patients with multiple myeloma (MM n=10). MFC data were compared to age-matched mononuclear cells of bone aspirates from non-malignant patients undergoing spine surgery (NMC n=10). Summary data showing the expression of the co-regulatory receptors on CD4+ T cells. P values were obtained by the ANOVA and Kruskal Wallis test. *P<0.05, **P<0.01, ***P<0.001, ****P<0.0001.
